# Alternating Therapy With Osilodrostat and Etomidate in Severe Ectopic Cushing’s Syndrome Complicated by Silent Bowel Perforation

**DOI:** 10.1155/crie/4689206

**Published:** 2026-06-09

**Authors:** Hery Mejia, Carmen Villabona, Renato Savian, Susana Barreiro Sacco, Alejandra Razzeto, Tanira Ferreira, Virginia Kondas, Sixto Arias, Elio Donna, Violet Lagari

**Affiliations:** ^1^ Division of Endocrinology, Diabetes and Metabolism, University of Miami, Miami, Florida, USA, miami.edu; ^2^ Division of Critical Care Medicine, University of Miami, Miami, Florida, USA, miami.edu; ^3^ Miami VA Healthcare System, Miami, Florida, USA

**Keywords:** colonic perforation, ectopic Cushing’s syndrome, etomidate therapy, hypercortisolemia complications, osilodrostat therapy, silent bowel perforation

## Abstract

Ectopic adrenocorticotropic hormone (ACTH)‐dependent Cushing’s syndrome (CS), ectopic ACTH secretion (EAS) is a rare condition caused by ACTH‐secreting neuroendocrine tumors (NETs), such as bronchial carcinoids. We report a 65‐year‐old woman with severe EAS complicated by bowel perforation. She presented with hypokalemia (K^+^ 2.3 mmol/L), metabolic alkalosis, resistant hypertension (180/110 mmHg), worsening diabetes (HbA1c 6.7%–9.1%), proximal muscle weakness, and 14 kg weight gain over 3 months. A silent sigmoid colon perforation required emergency resection and colostomy. Biochemical tests confirmed hypercortisolism (urine free cortisol [UFC], 1256 µg/24 h, plasma ACTH 175 pg/mL, and cortisol >40 µg/dL post‐dexamethasone). Imaging identified a 2.3 cm pulmonary nodule with mild uptake on Ga‐68 DOTATATE PET/CT. Bronchoscopic biopsy confirmed an ACTH‐positive low‐grade bronchial carcinoid tumor. Initial treatment with osilodrostat was interrupted due to acute illness and oral medication intolerance. Intravenous etomidate was employed in the ICU for rapid cortisol suppression, followed by resumption of osilodrostat after stabilization. Thoracoscopic lobectomy confirmed a low‐grade carcinoid tumor (Ki‐67 < 2%). Postoperatively, cortisol normalized, electrolytes stabilized, and HbA1c improved to 6.5%. This case highlights bowel perforation as a severe complication of EAS and underscores the importance of dynamic, alternating therapy with osilodrostat and etomidate, along with individualized surgical and medical management strategies.

## 1. Introduction

Ectopic secretion of adrenocorticotropic hormone (ACTH) is an uncommon condition, accounting for fewer than 17% of all cases of Cushing’s syndrome (CS) [1]. The estimated annual incidence is ~2–3 cases per million [2]. This condition arises from excessive hormone production due to small benign tumors or extensive metastatic disease [3]. Symptoms stem from elevated cortisol levels, leading to clinical manifestations such as hypokalemia, hyperglycemia, hypertension, weight gain, abdominal striae, easy bruising, hirsutism, menstrual disturbances, reduced libido, facial redness, and psychological disorders [3]. Diagnosing ectopic ACTH secretion (EAS) is challenging, necessitating dynamic hormonal assessments and advanced imaging techniques to pinpoint the ectopic source. However, in nearly 20% of cases, the origin remains elusive despite extensive investigations [4]. If untreated, EAS is associated with significant morbidity and mortality. Successfully identifying the source of ACTH production and surgically removing the lesion leads to disease remission in ~80% of cases and improves prognosis [5].

The diagnostic evaluation for ectopic CS involves confirming hypercortisolism through tests such as urine free cortisol (UFC), late‐night salivary cortisol (LNSC), or low‐dose dexamethasone suppression, followed by ACTH levels and the desmopressin stimulation test [6]. Computed tomography (CT) and MRI are used to localize ectopic ACTH sources [6]. When inconclusive, Gallium‐68‐labeled somatostatin receptor PET/CT (e.g., 68Ga‐DOTATATE PET/CT) provides enhanced sensitivity for detecting neuroendocrine tumor (NET) [6]. The management of ectopic ACTH‐secreting tumors involves various approaches, including surgical removal of the primary tumor, chemotherapy, radiation therapy, and the use of somatostatin analogs [2]. In addition to surgical treatment, medications including metyrapone, mitotane, and ketoconazole can lower circulating cortisol concentrations [7]. Osilodrostat inhibits 11β‐hydroxylase and prevents the conversion of 11‐deoxycorticosterone (DOC) to corticosterone and 11‐deoxycortisol (11‐DOF) to cortisol [8]. With a longer half‐life compared to metyrapone, osilodrostat enables more convenient once‐ or twice‐daily dosing, and demonstrates greater potency in suppressing 11β‐hydroxylase activity [8]. This case illustrates the diagnostic and management challenges of CS and highlights underrecognized but well‐known complications.

## 2. Case Presentation

A 65‐year‐old female with a history of type 2 diabetes mellitus, hypercholesterolemia, hypertension, and class I obesity presented with a worsening hyperglycemia. Over a period of 3 months, the patient’s hemoglobin A1c increased from 7.0% to 10.0% (reference: <5.7%), associated with a significant, unintentional weight loss of 10 kg. Subsequently, the patient developed easy bruising, fatigue, and progressive proximal muscle weakness. Laboratory tests demonstrated hypokalemia (2.1 mEq/L; reference: 3.5–5.0 mEq/L), metabolic alkalosis, hypomagnesemia (1.1 mg/dL; reference: 1.7–2.2 mg/dL), hypocalcemia (corrected calcium: 7.5 mg/dL; reference: 8.5–10.2 mg/dL), hypophosphatemia (1.1 mg/dL; reference: 2.5–4.5 mg/dL), and markedly elevated liver enzymes.

Vital signs at admission were blood pressure 135/88 mmHg, pulse 86 beats/min, oral temperature 98.2°F (36.8°C), respiratory rate 20 breaths/min, and oxygen saturation (SpO_2_) of 94% on room air. Physical examination revealed profound proximal muscle weakness and bruising of the extremities. There were no abdominal striae, supraclavicular, or supracervical fat pads (Table 1).

**Table 1:** Laboratory testing at admission.

Laboratory test	Result at admission	Reference range
Potassium (K)	2.1 mEq/L	3.5–5.0 mEq/L
Corrected calcium (CCa)	7.5 mg/dL	8.5–10.2 mg/dL
Phosphate (Phos)	1.1 mg/dL	2.5–4.5 mg/dL
Magnesium (Mg)	1.1 mg/dL	1.7–2.2 mg/dL
25‐Hydroxyvitamin D (25‐OH)	25 ng/mL	30–100 ng/mL
Glomerular filtration rate (GFR)	>90 mL/min	>90 mL/min
Parathyroid hormone (PTH)	229 pg/mL	15–65 pg/mL
Hemoglobin A1c	10%	<5.7%
Aspartate aminotransferase (AST)	202 U/L	10–40 U/L
Alanine aminotransferase (ALT)	276 U/L	7–56 U/L
Alkaline phosphatase (ALP)	372 U/L	44–147 U/L

### 2.1. Diagnostic Assessment

Initial laboratory testing revealed a morning serum cortisol level of 157 µg/dL (reference: 5–25 µg/dL), concurrent ACTH was 508 pg/mL (reference: 10–60 pg/mL), confirming ACTH‐dependence. Dynamic tests suggested EAS (DDAVP stimulation test and 8‐mg dexamethasone). A 24‐h UFC was also elevated at 258 µg/24 h (reference: 3.5–45 µg/24 h). A summary of the initial hypercortisolism workup is provided in Table 2.

**Table 2:** Initial hypercortisolism workup.

Test	Result	Reference range/protocol
Morning serum cortisol	157 µg/dL	5–25 µg/dL
ACTH concentration	508 pg mL	10–60 pg/mL
1‐mg dexamethasone suppression test (cortisol)	118 µg/dL	<1.8 µg/dL
8‐mg dexamethasone suppression test (cortisol)	148 µg/dL	<5 µg/dL
Desmopressin stimulation test	ACTH, 132 pg/mL baseline and 94 pg/mL at 60 min Cortisol: 125 µg/dL baseline and 139 at 60 min	Baseline ACTH and cortisol levels measured, followed by DDAVP 10 µg, with ACTH and cortisol reassessed at 60 min. Typical peak response: cortisol >20 µg/dL, ACTH increase of >50% from baseline.
Urine free cortisol	258 μg/24 h	3.5–45 μg/24 h

A pituitary MRI was negative for masses. A CT scan of the abdomen demonstrated bilateral adrenal hyperplasia (Figure 1), consistent with chronic ACTH stimulation. A CT of the chest revealed a 1.9 cm paraesophageal mass. A ^68^Ga‐DOTATATE PET/CT scan revealed a standardized uptake value (SUV) of 2.6, demonstrating mild radiotracer activity, which suggested a NET (Figure 2).

**Figure 1:**
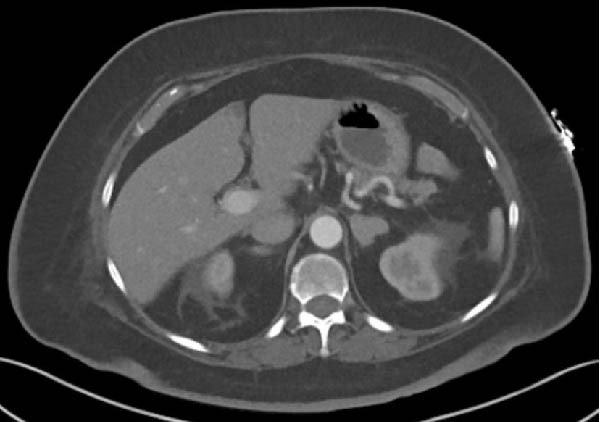
CT of the abdomen demonstrated bilateral adrenal hyperplasia, consistent with chronic ACTH stimulation.

**Figure 2:**
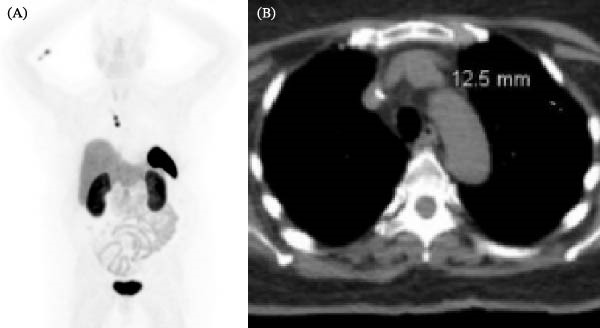
PET image (A) and CT image (B) demonstrating a paraesophageal mass with corresponding mild radiotracer uptake.

The patient presented in a severely ill state, warranting initiation of medical management to control hypercortisolism prior to confirmation by fine‐needle aspiration (FNA) biopsy. A FNA biopsy of the paraesophageal mass was performed to determine the source of EAS. Cytological analysis confirmed a low‐grade NET, consistent with a typical carcinoid tumor producing ectopic ACTH (Figure 3). This confirmed the diagnosis of EAS secondary to a bronchial carcinoid tumor.

**Figure 3:**
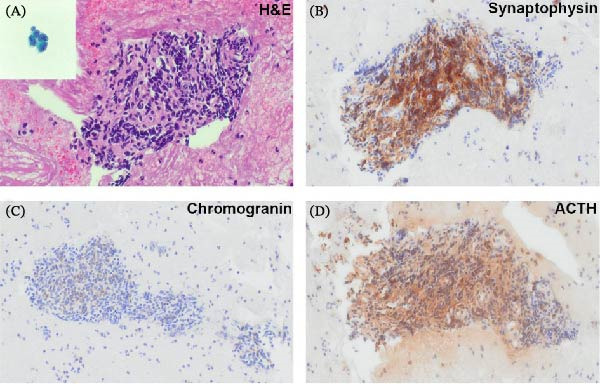
The pathology slides demonstrate a low‐grade neuroendocrine tumor with small to medium‐sized cells on H&E stain (A), strong synaptophysin positivity (B) confirming neuroendocrine differentiation, focal chromogranin positivity (C), and strong ACTH positivity (D), confirming the tumor as the ectopic source of ACTH production in this case of ACTH‐dependent Cushing’s syndrome.

### 2.2. Treatment

Osilodrostat, an oral, selective 11β‐hydroxylase inhibitor, was initiated at 10 mg twice daily. Cortisol levels decreased from 157 to 50 µg/dL within 3 days. Despite initial improvements, the patient developed a colonic perforation and abscess. Emergent surgery was required, and a sigmoid colectomy with temporary ileostomy was performed to stabilize the patient. The postoperative course was complicated by hemodynamic instability, necessitating the use of vasopressors and mechanical ventilation. During this time, the patient was still taking osilodrostat. Hydrocortisone was initiated at 25 mg every 8 h to prevent adrenal insufficiency/crisis. With the patient intubated and NPO, a single dose of osilodrostat was administered via a nasogastric (NG) tube but was soon discontinued given the potential for lack of absorption. As cortisol levels continued to rise, reaching 56 µg/dL, an etomidate infusion was initiated at 0.02 mg/kg/h and plasma cortisol levels decreased to 17 µg/dL within 5 days. Etomidate was further titrated up to suppress endogenous hypercortisolemia, and hydrocortisone was administered to prevent adrenal insufficiency.

Throughout these initial weeks, the patient experienced acute colon perforation, sepsis caused by *Enterococcus faecium*, acute respiratory distress syndrome (ARDS), *Pneumocystis jirovecii* pneumonia despite baseline coverage with Bactrim, and Herpes simplex virus infection. Once able to resume oral intake, etomidate was discontinued, and osilodrostat was reintroduced at 15–20 mg twice daily, and biochemical suppression was confirmed with 2UFC measurements, which decreased from 258 µg/24 h to 12 µg/24 h within 6 days of therapy. Figure 4 illustrates the temporal changes in cortisol levels across two comparisons: cortisol levels relative to osilodrostat dosing and cortisol levels relative to etomidate dosing. Figure 5 illustrates the impact of osilodrostat and etomidate on cortisol levels over ~80 days.

**Figure 4:**
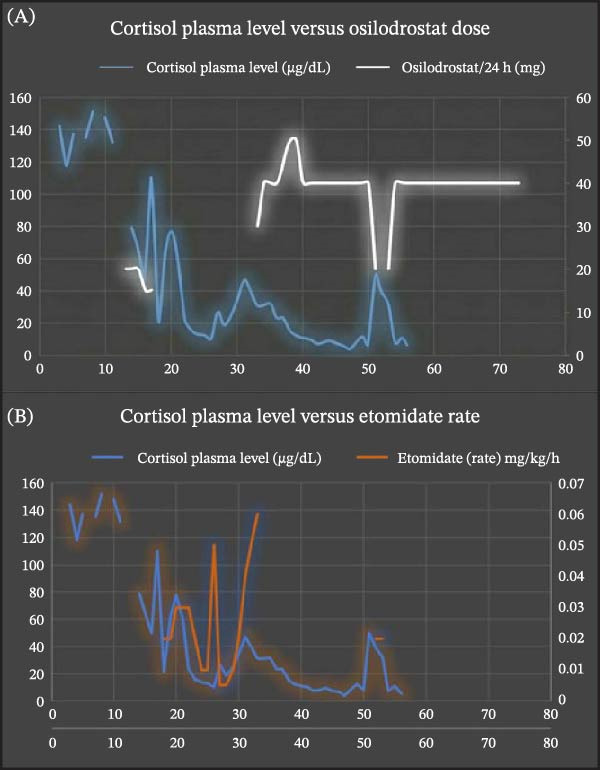
Comparison of cortisol levels in response to osilodrostat and etomidate. This figure shows plasma cortisol levels over time in response to two medications. The top graph (A) illustrates cortisol levels (µg/dL) relative to osilodrostat dose (mg/24 h), while the bottom graph (B) shows cortisol levels relative to etomidate infusion rate (mg/kg/h). These drugs were administered in an alternated way, with minimal or no overlap, to allow a clear comparison of their effects on cortisol suppression.

**Figure 5:**
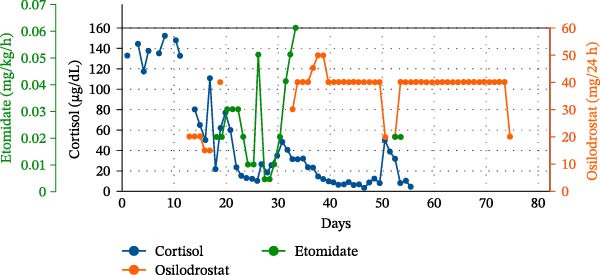
Comparative impact of osilodrostat and etomidate on cortisol levels over time. This graph shows cortisol levels (blue) and the administration of osilodrostat (orange) and etomidate (green) across 80 days. Cortisol fluctuates initially but decreases and stabilizes after day 30. Osilodrostat is administered consistently with some adjustments, while etomidate is given intermittently, shown by sharp peaks. The graph highlights each medication’s effect on cortisol suppression and dosing patterns over time.

The patient underwent a right robotic‐assisted thoracoscopic lobectomy with complete thoracic lymphadenectomy to remove a 2.5 cm ACTH‐producing NET in the right lower lobe. She had an extensive lymph node dissection (stations 2R, 4R, 7, 8, 9, and 10). Histopathologic examination confirmed a low‐grade NET (typical carcinoid) with no evidence of lymphovascular invasion or nodal metastasis. Tumor was classified as pT1cN0. Hypokalemia resulting from cortisol excess and osilodrostat use was managed with spironolactone 50–100 mg twice daily, in combination with potassium supplementation. QTc intervals were also monitored. Figure 5 summarizes the patient’s clinical course, highlighting key diagnostic findings, medical, and surgical interventions.

### 2.3. Outcome and Follow‐Up

Following surgical resection of an ACTH‐producing right lower lobe NET, hydrocortisone was tapered to 20 mg in the morning and 10 mg in the evening, and a plasma cortisol of 8.9 µg/dL was reached. As the patient’s condition stabilized, the patient progressed sufficiently to transition to a rehabilitation facility for continued recovery. Her 8 a.m. outpatient serum cortisol level was stable at 9 μg/dL, allowing for discontinuation of hydrocortisone. Follow‐up assays, including LNSC and UFC, showed low cortisol levels (<0.03 μg/dL and 8 μg/24 h, respectively), confirming remission.

## 3. Discussion

Severe hypercortisolism is a life‐threatening condition that occurs infrequently (<5% cases) in patients with CS [9]. Due to the life‐threatening bowel perforation and unclear source of ACTH production at presentation, the patient required aggressive treatment during the diagnostic work‐up until the patient was stable enough for surgery. Due to the life‐threatening bowel perforation, and unclear source of ACTH production at presentation, the patient required aggressive treatment during the diagnostic work‐up until the patient was stable enough for surgery. Overall, data are limited regarding this complication [10]. One case series described six patients with both CS and perforation of a colonic diverticulum [10]. Demographically, patients were older, with two of the four patients having atypical presentations and minimal abdominal symptoms; all patients had severe hypercortisolemia [10]. Early diagnosis of intestinal perforation is critical, as it has a high mortality rate of about 39%. The current and previously reported cases suggest that clinicians should maintain a low threshold for suspecting bowel perforation in severely hypercortisolemic patients, regardless of the cause of hypercortisolism, age, or sex. The absence of typical abdominal signs does not rule out the diagnosis [10].

Severe hypercortisolism, which leads to an immunocompromised state, increases the risk of diverticulitis and perforation [10]. Intestinal perforation typically occurs due to severe inflammation and necrosis of the bowel wall [10]. In the patients described, despite evidence of bowel inflammation on imaging and surgical inspection, they did not show typical systemic signs of inflammation, such as fever or significant leukocytosis, and their abdominal symptoms were less severe than expected [10]. This atypical presentation in hypercortisolemic patients may be due to glucocorticoids inhibiting early inflammatory responses [10]. If this hypothesis is correct, waiting for typical abdominal signs could delay treatment [10]. Glucocorticoids are known to suppress collagen and DNA synthesis in the skin, which may also affect the intestinal mucosa, leading to tissue fragility and increased risk of perforation [10]. Other potential mechanisms for gastrointestinal perforation in CS include hypokalemia‐induced constipation, decreased turnover of intestinal cells, and reduced bowel lymphoid tissue, which could make the gut more vulnerable to bacterial invasion [10].

While various drugs were available, our patient required a potent medication with a favorable safety profile, given the critical condition. Ketoconazole is known for causing hepatitis and was therefore contraindicated in this patient with elevated liver enzymes (AST 202 U/L; ALT 276 U/L) [11]. Osilodrostat, a potent drug that inhibits both 11‐beta hydroxylase and aldosterone synthetase, was chosen as the first‐line therapy [12]. Results from two Phase III trials (LINC 3 and LINC 4) demonstrated that osilodrostat achieved rapid and sustained decreases in UFC levels in the majority of patients with Cushing’s disease [13]. Standard dosing starts at 2 mg orally twice daily (4 mg/day), up to a maximum of 60 mg/day, but higher doses may be used in severe cases [8, 14]. The patient was quickly titrated from 10 to 20 mg twice daily.

After just 3 days of treatment with Osilodrostat at a dose of 10 mg twice daily, the patient’s plasma cortisol levels dropped from 139 to 50 µg/dL. In a multicenter retrospective study on the use of osilodrostat in EAS, two‐thirds of patients were treated with a block‐and‐replace regimen at the last follow‐up, confirming the effectiveness of this approach in managing severe hypercortisolism [15]. During the patient’s hospitalization, corticosteroid therapy was administered on multiple occasions, guided by plasma and urine cortisol levels and the patient’s clinical condition. Cortisol measurements should be performed by mass spectrometry, to avoid cross‐reactivity with cortisol precursors like 11‐deoxycortisol, which can rise during osilodrostat treatment and falsely elevate cortisol readings [8]. Our lab utilized liquid chromatography‐tandem mass spectrometry (LC‐MS/MS), ensuring reliable cortisol levels. The plasma cortisol level decreased from 139 to 50 within 3 days while the patient was on osilodrostat 10 mg PO twice daily, and it was later confirmed with a UFC test of 12 mg/24 h while the patient was on a dose of 20 mg PO twice daily.

With the patient unable to take oral medications, osilodrostat was discontinued, and etomidate infusion was started. Etomidate inhibits glucocorticoid synthesis (including cortisol, corticosterone, and 18‐hydroxycorticosterone) by 11β‐hydroxylase inhibition [16]. Gastrointestinal tract perforation is a known complication of CS, which limits the use of oral steroidogenesis inhibitors, including ketoconazole, metyrapone, and osilodrostat [16]. In such cases, etomidate remains the only steroidogenic inhibitor available for intravenous administration [16]. Etomidate dosing protocols for cortisol suppression in CS have been described across various studies and guidelines. The Endocrine Society recommends a loading dose of 3–5 mg followed by continuous infusion at 0.03–0.10 mg/kg/h, with cortisol monitoring every 4–6 h and a target serum cortisol of 10–20 μg/dL [17]. Fleseriu et al. [8] further stratified dosing into low‐dose (0.04–0.05 mg/kg/h) for partial blockage, high‐dose (0.5–1 mg/kg/h) for complete blockade with hydrocortisone replacement, and very low dose (0.025 mg/kg/h) for non‐ICU settings. Preda et al. [18] provided a comprehensive review of etomidate protocols, emphasizing the importance of ICU monitoring and individualized dose titration. More recently, Constantinescu et al. [19] demonstrated that very low‐dose etomidate (media 0.025 mg/kg/day) administered outside the ICU was effective and safe, with no patients requiring hydrocortisone supplementation, in contrast to high‐dose ICU protocols where all patients developed cortisol insufficiency. Carroll et al. [20] and Soh et al. [21] also reported on etomidate infusion protocols for severe hypercortisolism.

Our patient had a bowel perforation and became critically ill during the hospital course, requiring an IV agent to control life‐threatening hypercortisolism. The administration of etomidate can be associated with significant side effects, including the risk of adrenocortical insufficiency, especially when not accompanied by glucocorticoid replacement therapy [16]. Our approach of alternating between osilodrostat and etomidate is only one of several reports in the medical literature [22, 23]. Dzialach et al. [23] evaluated low‐dose, lipid‐formulated etomidate infusion in seven patients with severe CS due to EAS. Continuous etomidate infusion (starting at 0.01–0.02 mg/kg/h without a loading bolus) achieved target cortisol levels in all patients within a median of 30 h, with median serum cortisol decreasing from 101.9to 19.5 μg/dL after 72 h. Treatment was administered outside the ICU with minimal sedation‐related adverse effects, and four patients were successfully transitioned to osilodrostat monotherapy using a combined etomidate‐osilodrostat bridging strategy. The authors conclude that low‐dose etomidate remains a valuable option for rapid hypercortisolemia control in SCS, particularly when oral steroidogenesis inhibitors are not immediately available, and that the lipid formulation may be preferable to propylene glycol‐based formulations to avoid solvent‐related toxicity [23]. Krzystek et al. [24] further corroborated this approach, describing the combined use of etomidate and osilodrostat in severe hypercortisolemia. While Dzialach et al. [23] and Krzystek et al. [24] reported concurrent etomidate‐osilodrostat administration as a planned bridging strategy, our case illustrates a sequential alternating approach necessitated by clinical circumstances that precluded the use of oral medications during acute illness, followed by resumption of osilodrostat after stabilization. Both strategies emphasize the complementary pharmacologic profiles of these agents: etomidate for rapid IV cortisol suppression and osilodrostat for sustained oral maintenance therapy.

Compared with other therapeutic approaches, etomidate offers the advantage of rapid onset and parenteral administration, making it uniquely suited for patients with gastrointestinal compromise or altered consciousness. However, its use requires close monitoring due to the risk of adrenal insufficiency and the need for ICU‐level or intensive supervision in many protocols. Oral agents such as osilodrostat and metyrapone offer the convenience of outpatient management and dose titration but are limited by gastrointestinal absorption requirements and slower onset of action. Ketoconazole, while effective, carries hepatotoxicity risks and drug interaction concerns. Bilateral adrenalectomy provides definitive cortisol control but is invasive and results in permanent adrenal insufficiency. The combined etomidate‐osilodrostat strategy leverages the rapid action of etomidate while transitioning to sustainable oral therapy, representing a practical bridge in severe cases. After achieving cortisol control with steroidogenesis blockade, our patient was placed on hydrocortisone (20 mg in the morning, 10 mg in the afternoon) to prevent adrenal insufficiency.

Excess glucocorticoids lead to mineralocorticoid receptor activation, vasopressin antagonism, and increased angiotensinogen synthesis, resulting in renal potassium wasting, sodium reabsorption, and hypertension, which are common features of EAS [25, 26]. Hypokalemia is further compounded by osilodrostat use, as accumulation of adrenal steroid precursors with mineralocorticoid properties enhances potassium loss; the LINC 3 and LINC 4 trials reported hypokalemia as a notable adverse event often requiring supplementation [27]. Our patient exhibited profound hypokalemia (serum potassium 2.1 mEq/L), which was managed with potassium supplementation and spironolactone (100–300 mg/day). Spironolactone also contributed to improved blood pressure control, alongside angiotensin‐converting enzyme inhibitors, angiotensin receptor blockers, and β‐blockers as needed.

The rapid onset of hypercortisolism seen in EAS often leads to depression, anxiety, and even psychosis, reported in up to 50% of patients. Severe cases may require haloperidol or other antipsychotics [28]. In our case, the patient’s depression and anxiety were managed with selective serotonin reuptake inhibitors and benzodiazepines.

Hypercortisolism drives hyperglycemia through multiple mechanisms, including increased hepatic gluconeogenesis, insulin resistance, and impaired pancreatic beta‐cell function [2, 29]. Prior to hospitalization, the patient’s diabetes regimen consisted of basal‐bolus insulin therapy, metformin, and a GLP‐1 agonist. During hospitalization, basal‐bolus insulin therapy continued, and upon discharge, the patient was transitioned to metformin monotherapy.

## 4. Conclusion

This case demonstrates the challenging diagnosis and management of severe ACTH‐dependent ectopic CS, complicated by silent colonic perforation and the need to alternate between osilodrostat and etomidate to control hypercortisolemia. The clinical course was further complicated by *Pneumocystis jirovecii* pneumonia, ARDS, and sepsis, underscoring the importance of coordinated care across endocrinology, critical care, infectious disease, and surgery. Careful titration of corticosteroids was essential to avoid adrenal insufficiency while maintaining adequate cortisol suppression. Physical rehabilitation played a key role in managing hypercortisolism‐induced myopathy and promoting recovery. This case offers valuable insights into the treatment of refractory CS, demonstrating the need for precise treatment strategies, continuous monitoring, and effective multidisciplinary collaboration to achieve favorable outcomes.

## 5. Learning Points

### 5.1. Alternating Osilodrostat and Etomidate Use in Severe Cushing’s

The alternating use of osilodrostat and etomidate for cortisol suppression represents a novel approach to managing hypercortisolism in the setting of a dynamic clinical status, where the use of one or the other therapy may be limited by ongoing clinical changes.

### 5.2. Frequent Dynamic Adjustments in Cortisol Therapy

Dynamic, real‐time monitoring of cortisol levels, with frequent adjustments to therapeutic agents, underscores the need for personalized treatment in cases of unstable hypercortisolism, ensuring precise control in critical settings.

### 5.3. Electrolyte Imbalances as a Predominant Presentation

This case highlights the rare presentation of EAS, where severe electrolyte disturbances such as hypokalemia and metabolic alkalosis were the primary features, rather than typical Cushingoid signs only.

### 5.4. Mild Ga‐68 DOTATATE PET/CT Uptake in NET

Mild radiotracer uptake on PET‐DOTATATE imaging, as seen in this patient, can still indicate significant pathology such as a bronchial carcinoid tumor, emphasizing the importance of clinical correlation in cases of EAS.

### 5.5. Osilodrostat as a Key Agent in the Inpatient Management of Hypercortisolemia Crisis

The use of osilodrostat in the inpatient setting for acute hypercortisolemia showcases its role as an effective steroidogenesis inhibitor, particularly in the context of a hypercortisolism emergency where rapid control of cortisol levels is essential but must be balanced with the need for real‐time therapeutic adjustments.

## Author Contributions

Hery Mejia contributed to the case description, diagnostic assessment, and management. Renato Savian contributed to the introduction, case description, diagnostic assessment, management, conclusion, and organization of tables and figures. Susana Barreiro Sacco and Alejandra Razzeto were involved in the treatment and complications sections. Violet Lagari and Carmen Villabona provided critical review and oversight across the manuscript. Violet Lagari had full access to all of the data in this study and takes complete responsibility for the integrity of the data and the accuracy of the data analysis.

## Funding

No public or commercial funding was received for this manuscript.

## Disclosure

All authors have read and approved the final version of the manuscript. This manuscript was presented as an abstract at the Endocrine Society Meeting in 2025 [30].

## Consent

Written informed consent was obtained from the patient for the publication of clinical details and/or images included in this manuscript, in accordance with institutional guidelines and ethical standards.

## Conflicts of Interest

The authors declare no conflicts of interest.

## Data Availability

The data that support the findings of this study are available upon request from the corresponding author. The data are not publicly available due to privacy or ethical restrictions.
